# Modulating γ‐secretase activity to make precision medicine for AD

**DOI:** 10.1002/alz.090507

**Published:** 2025-01-09

**Authors:** Lucia Chávez‐Gutiérrez

**Affiliations:** ^1^ VIB‐KULeuven, Leuven, Vlaams Brabant Belgium

## Abstract

Gamma‐secretases play a pivotal role in the generation of Aβ peptides. Mutations in these enzymes that cause early‐onset, autosomal dominant AD shift Aβ production towards generation of longer peptides. We have recently shown that the mutation‐induced shifts in the ratio of short‐to‐long Aβ peptides not only inform about mutation pathogenicity but also allow experimental prediction of the age at dementia onset. This observation argues in favor of therapeutic strategies that shift Aβ production towards the generation of shorter and non‐toxic Aβ peptides.

By stabilizing GSEC‐APP interactions and activating the GSEC processive cleavage of APP, GSEC modulators (GSMs) have the potential to selectively reduce the levels of longer and toxic Aβ peptides, without hindering essential cellular processes that depend on the overall GSEC proteolytic activity. GSMs thus offer a nuanced approach to prevent, and potentially slow down, amyloid‐driven neurodegenerative cascades in AD therapy. However, thorough mechanistic investigation of the modulatory actions of GSMs on GSEC activities and biology is paramount to unlock the full therapeutic potential of GSMs in the clinic.

I will present and discuss our latest mechanistic studies of GSEC (dys)function and its modulation by GSMs acting as GSEC‐APP stabilizers.

